# Network pharmacology and molecular docking reveal the immunomodulatory mechanism of rhubarb peony decoction for the treatment of ulcerative colitis and irritable bowel syndrome

**DOI:** 10.3389/jpps.2023.11225

**Published:** 2023-05-25

**Authors:** Leilei Zhai, Weiming Yang, Dianrong Li, Wei Zhou, Min Cui, Ping Yao

**Affiliations:** ^1^ Graduate School, Xinjiang Medical University, Urumqi, China; ^2^ Department of Gastroenterology, The First Affiliated Hospital of Xinjiang Medical University, Urumqi, China; ^3^ Department of Nephrology, The Children’s Hospital, Zhejiang University School of Medicine, National Clinical Research Center for Child Health, Hangzhou, China

**Keywords:** ulcerative colitis, network pharmacology, molecular docking, irritable bowel syndrome, rhubarb peony decoction

## Abstract

**Background:** Ulcerative colitis (UC) and irritable bowel syndrome (IBS) share various similarities in clinical symptoms, pathogenesis, and treatment. UC concurrent IBS tends toward more severe symptoms and worse prognosis, and promising feasible therapies for the overlapping symptoms remains a challenge. Rhubarb peony decoction (RPD) is a well-known traditional Chinese medicine that has been widely applied in treating UC. RPD may exert extensive therapeutic effects on both IBS and UC. However, the common mechanism of its treatment remains unclear. We aimed to assess the potential pharmacological mechanism of RPD in the treatment of overlapping IBS and UC.

**Methods:** The active components and targets of RPD were retrieved from ETCM, TCMSP, BATMAN-TCM, and TCM databases. The disease targets were screened by searching the DrugBank, OMIM, TTD, and PharmGKB databases. PPI network analysis was performed and visualized via the STRING platform and Cytoscape software. GO and KEGG enrichment analyses of the hub genes of RPD were predicted to elucidate the potential molecular mechanism. Subsequently, molecular docking was carried out to verify the combination of active compounds with core targets.

**Results:** By integrating all targets of RPD and disease, a total of 31 bioactive ingredients were identified including quercetin, kaempferol, aloe-emodin, beta-sitosterol, and (+)-catechin, etc. JUN, TP53, MAPK1, RELA, MYC, and ESR1 were explored as potential therapeutic targets among 126 common drug-disease-related targets. They were enriched in the AGE-RAGE signaling pathway in diabetic complications, as well as the NF-kappa B signaling pathway and MAPK signaling pathway. Additionally, some active ingredients were identified as candidates for binding to the hub targets via molecular docking, further suggesting their anti-inflammatory and antioxidative properties.

**Conclusion:** RPD may exert the overall treatment effect for UC and IBS overlap syndrome via the biological mechanism of “multi-ingredients, multi-targets, and multi-pathways” on inflammation, oxidative stress, immune, oncogenicity, and gut microbiota dysbiosis.

## Introduction

Ulcerative colitis (UC) is a chronic idiopathic inflammatory disease that affects the colon, characterized by recurrence and remission of mucosal inflammation. Mucosal inflammation begins in the rectum and continues proximally to the colon [1]. The morbidity of UC is increasing in developing countries, thereby constituting a major public health problem [2]. Irritable Bowel Syndrome (IBS) is one of the most prevalent functional gastrointestinal disorders [3]. It affects approximately 10%∼ 25% of adults worldwide and occurs primarily in women [4, 5]. IBS typically manifests as a chronic and relapsing condition, characterized by several intestinal symptoms including abdominal pain, bloating, a sensation of incomplete evacuation, and altered bowel habits [5, 6]. It has become a healthcare burden on society due to severe psychological state and quality of life (QoL) damage [7]. UC and IBS appear to be quite separate entities, but they do share similarities in clinical symptoms and pathogenesis, even for treatment. Common etiology includes altered gut permeability, gut microbiota dysbiosis, mucosal immune responses, psychological factors, and abnormalities of enteric nerve structure and function [8–10]. Evidence has revealed that around 36% of UC patients have persistent IBS-like symptoms containing abnormalities of sensation, motility, and gut microbiota [11]. Current treatments for IBS include tachykinin, histamine receptor antagonists, and dietary therapy [12]. Therapeutic options for UC include 5-aminosalicylic acid, corticosteroids, anti-TNF agents, and JAK inhibitors [13]. Due to the similarity in pathogenesis, the same treatment may be used for IBS and mild UC patients, including Mesalazine, microecological preparations, and dietary therapy. With the prolongation of western medicine treatment time, distinct degrees of side effects will occur to affect adherence to the treatment process and thus reduce efficacy.

Traditional Chinese medicine (TCM) has been considered an alternative therapy for UC and IBS. TCM is known for its mild nature and has fewer side effects than western medicines. Rhubarb peony decoction (RPD) is a well-known TCM formula, first described in the “Golden Chamber Synopsis” written by the Chinese physician Zhongjing Zhang in the Chinese Eastern Han dynasty, and has more than 2000 years of clinical application history in China. RPD is composed of five herbs: da huang (*Rhei Radix et Rhizoma*), tao ren (*Persicae semen*), dong gua zi (*Benincasae semen*), dan pi (*Moutan Cortex*), and Mirabilite (a mineral substance, Na_2_SO_4_·10H_2_O). RPD is widely used to treat intestinal carbuncle in China. So far, its novel and effective pharmacological effects on UC have been repeatedly discovered. Luo et al. confirmed that RPD could significantly alleviate the pathological damage in the colon of UC mice by reducing the proportion of Thl7 cells and inflammatory cytokine mediators [14]. Moreover, its major active ingredient, 6-methyl-1,3,8-trihydroxyanthraquinone with the commercial name of Emodin appears to have multiple health benefits in the treatment of a host of diseases, such as immune-inflammatory abnormality, bacterial infections, and tumor progression [15].

**FIGURE 1:**
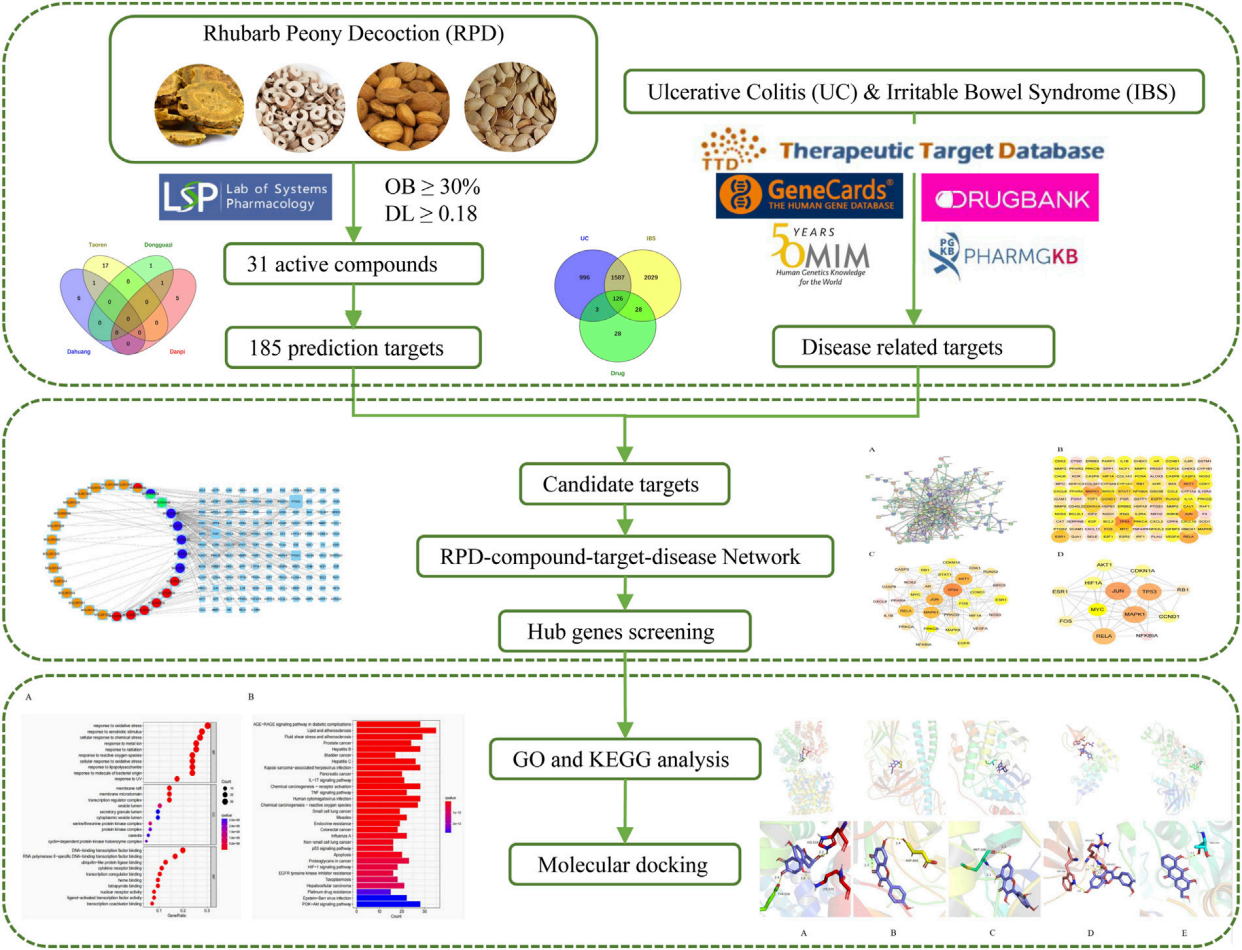
The flowchart of underlining pharmacological mechanisms of RPD against UC and IBS.

Consistent with UC, spleen deficiency and liver stagnation are the root causes of IBS. We speculate that RPD can not only exert significant effects on UC but also IBS. There is a lack of study on the common biological mechanism of RPD in treating IBS and UC. The explanation of the scientific connotation of “the same treatment of different diseases” is one of the significant contents of TCM modernization. Therefore, this study attempted to undertake network pharmacology to further explore the underlying core targets and pathways of the overall treatment effect of RPD against both IBS and UC (Figure 1). Further, this study aimed to provide a reliable reference value for subsequent research including experimental verification and new drug development, as well as for new clinical application.

## Materials and methods

### Screening of active components and relative targets in RPD

The active chemical components of five herbs in RPD were retrieved from four public databases including the Traditional Chinese Medicine Systems Pharmacology Database and Analysis Platform(TCMSP, https://tcmspw.com/tcmsp.php) [16], the Bioinformatics Analysis Tool for Molecular mechanism of Traditional Chinese Medicine (BATMAN-TCM, http://bionet.ncpsb.org.cn/batman-tcm/) [17], the Encyclopedia of Traditional Chinese Medicine (ETCM, http://www.tcmip.cn/ETCM/index.php/Home/Index/index.html) [18], and Traditional Chinese Medicine Database@Taiwan (TCM Database@Taiwan, http://tcm.cmu.edu.tw/) [19]. All of these platforms integrate large-scale authoritative data, including absorption, distribution, metabolism, excretion index scores (ADME parameters), target prediction, and chemical properties. According to the optimal toxicokinetic ADME rules in the TCMSP database, the above TCM compounds were screened as candidate target compounds. Oral bioavailability (OB) ≥ 30% and drug-like (DL) ≥ 0.18 were established as the preset criteria. The collected targets were imported into UniProt (http://www.uniprot.org/) for annotating gene names.

### The retrieval of relative disease targets of UC and IBS

In addition, disease-related targets were retrieved from five public databases via the keywords “ulcerative colitis” and “irritable bowel syndrome”: GeneCards (https://www.genecards.org/), Online Mendelian Inheritance in Man (OMIM, https://omim.org/), PharmGKB (https://www.pharmgkb.org/), Therapeutic Target Database (TTD, http://db.idrblab.net/ttd/), and DrugBank (https://go.drugbank.com/). Finally, the common targets of UC and IBS were obtained by merging and eliminating the repeated targets of each disease database.

### Constructing the database of intersecting drug-disease target genes

We hypothesized that cross-targeting compounds may be common therapeutic components affecting UC and IBS. Genes that were irrelevant or mismatched between the disease target and RPD were removed. By integrating all targets, the cross-target genes were extracted to construct the assembled target gene library for the following research.

### Constructing the protein-protein interaction (PPI) network

The PPI network was constructed and clarified by STRING (https://string-db.org/) [20]. “*multiple proteins*” was chosen, “*Homo sapiens*” was set for protein species, and “*highest confidence (0.9)*” was the minimum interaction threshold. The PPI network was generated to visualize the complex connections. In addition, both active ingredients and corresponding targets were imported into Cytoscape 3.6.1 to further elucidate the interactions of the common target genes in the treatment of UC and IBS with RPD. The CytoNCA plugin was used to calculate the topological parameters of nodes to provide an in-depth analysis of the attributes of nodes in the interactive network. Six main reference parameters were calculated including betweenness centrality (BC), closeness centrality (CC), degree centrality (DC), eigenvector centrality (EC), network centrality (NC), and local average connectivity (LAC). Higher quantitative values of parameters indicate the greater significance of nodes in the network. Target nodes with all six parameters higher than the corresponding median values in the PPI network were selected to construct a new PPI network. The node selection criteria were repeated to obtain the core PPI network.

### Enrichment analysis of GO and KEGG

Gene Ontology (GO) term enrichment and Kyoto Encyclopedia of Genes and Genomes (KEGG) pathway enrichment analysis were carried out via the R package including “colorspace,” “clusterProfiler,” “dose,” “stringi,” “enrichplot,” and “ggplot2” which were installed in software R 4.2.1. The function “enrichGO” was carried out for GO enrichment analysis, and the database was org. Hs .eg. db (DOI: 10.18129/b9.bioc.org.Hs.eg.db), the “enrichKEGG” function was applied for KEGG enrichment analysis, and the relative database was the KEGG database (https://www.kegg.jp/), respectively. It was determined that the functional terms and pathways with q-values (enrichment score) <0.05 were regarded as statistically significant and retained. The results were sorted according to the q-value, and the top 20 results were visualized as barplot or bubble diagram.

### Verification of ingredient–target interaction via molecular docking

The key targets were molecularly docked to the active ingredients for the primary validation. The three-dimensional(3D) structures of key target proteins were downloaded from the Protein Database Bank (https://www.rcsb.org/), and 2D molecular structures of ligands were derived from the PubChem database (https://pubchem.ncbi.nlm.nih.gov/), respectively [21]. Chem3D software was utilized to convert ligands into a 3D structure, obtaining the most stable molecular conformation with minimized energy by optimization module. The target protein was presented as a receptor and the water molecule was added with nonpolar hydrogen. The core bioactives and selective proteins were docked by AutoDockVina software and visualized by PyMOL software [22]. The lowest binding energy was regarded as the best-docked conformation, and spontaneous binding occurred while the energy below −5 kJ/mol [23]. A heatmap plot was drawn to visualize these values in R.

## Results

### Active components and target selection of RPD

**FIGURE 2:**
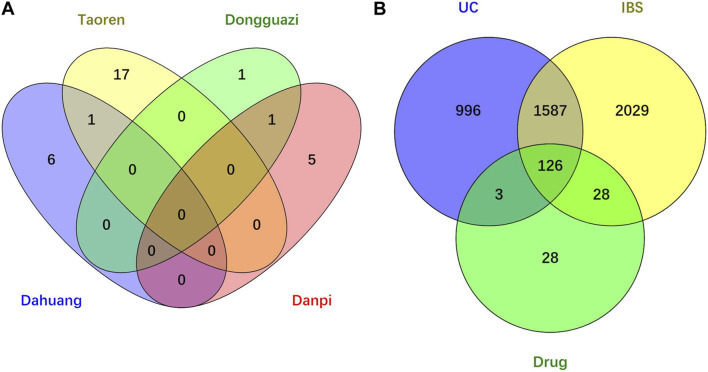
Venn diagram of drug-disease intersection target of RPD for UC and IBS. **(A)** Core compounds of four herbs from RPD. **(B)** The common targets of RPD-UC-IBS.

Through searching the TCMSP and other online databases to obtain the compounds and prediction targets of RPD. Mirabilite (Na_2_SO_4_·10H_2_O) is a kind of inorganic compound that exerts effects on drug absorption by promoting small intestine movement [24]. After performing ADME screening (OB≥30, DL≥0.18), 31 active ingredients of the main medicine in this prescription were screened (containing 7 types in da huang, 6 types in dan pi, 18 types in tao ren, and 2 types in dong gua zi) (Figure 2A). Using the related target prediction technology in each software to screen the prediction targets of the above-mentioned active ingredients, the repeated targets were eliminated, and finally a total of 185 herbal prediction targets were obtained (Figure 2B) (Table 1, Supplementary Table S1).

**TABLE 1:** Active compounds of RPD.

Drug	Mol Id	Molecule name	OB (%)	DL
dan pi	MOL000211	Mairin	55.38	0.78
dan pi	MOL000422	kaempferol	41.88	0.24
dan pi	MOL000492	(+)-catechin	54.83	0.24
dan pi	MOL007374	5-[[5-(4-methoxyphenyl)-2-furyl] methylene] barbituric acid	43.44	0.3
dan pi	MOL000098	quercetin	46.43	0.28
dan pi/dong gua zi	MOL000359	sitosterol	36.91	0.75
dong gua zi	MOL000449	Stigmasterol	43.83	0.76
da huang	MOL002235	EUPATIN	50.8	0.41
da huang	MOL002268	Rhein	47.07	0.28
da huang	MOL002281	Toralactone	46.46	0.24
da huang	MOL002297	Daucosterol_qt	35.89	0.7
da huang	MOL000358	beta-sitosterol	36.91	0.75
da huang	MOL000471	aloe-emodin	83.38	0.24
da huang	MOL000096	(-)-catechin	49.68	0.24
da huang/tao ren	MOL001358	gibberellin 7	73.8	0.5
tao ren	MOL001323	Sitosterol alpha1	43.28	0.78
tao ren	MOL001328	2,3-didehydro GA70	63.29	0.5
tao ren	MOL001329	2,3-didehydro GA77	88.08	0.53
tao ren	MOL001340	GA120	84.85	0.45
tao ren	MOL001342	GA121-isolactone	72.7	0.54
tao ren	MOL001344	GA122-isolactone	88.11	0.54
tao ren	MOL001349	4a-formyl-7alpha-hydroxy-1-methyl-8-methylidene-4aalpha,4bbeta-gibbane-1alpha,10beta-dicarboxylic acid	88.6	0.46
tao ren	MOL001351	Gibberellin A44	101.61	0.54
tao ren	MOL001352	GA54	64.21	0.53
tao ren	MOL001353	GA60	93.17	0.53
tao ren	MOL001355	GA63	65.54	0.54
tao ren	MOL001360	GA77	87.89	0.53
tao ren	MOL001361	GA87	68.85	0.57
tao ren	MOL001368	3-O-p-coumaroylquinic acid	37.63	0.29
tao ren	MOL000296	hederagenin	36.91	0.75
tao ren	MOL000493	campesterol	37.58	0.71

OB, oral bioavailability; DL, drug-likeness.

### Screening disease targets for UC and IBS

We searched the DisGeNET, Drugbank, OMIM, TTD, and PharmGKB databases to obtain the known target genes related to UC and IBS, respectively. Merging each database and deleting the duplicates, we retrieved 3,770 disease targets for IBS and 2,712 disease targets for UC (Supplementary Figures S1, S2). A total of 1713 (63.2% of UC, 45.4% of IBS) disease genes overlap among them, demonstrating they do share similarities in critical pathogenesis. PRD could exert common effects on these two diseases with the explanation of the scientific connotation of “the same treatment of different diseases.” By merging with RPD-related targets, we obtained 154 IBS-related therapeutic targets and 129 UC-related therapeutic targets. Finally, 126 intersecting target genes were considered as the predicted targets for subsequent studies (Table 2). OmicShare online tool was used to construct a Venn diagram for intuitive visual presentation **(** Figure 2B).

**TABLE 2:** The intersection targets of RPD and diseases related to UC and IBS.

No.	Gene	No.	Gene	No.	Gene	No.	Gene
1	ABCG2	33	COL3A1	65	IFNG	97	PLAU
2	ACHE	34	CTSD	66	IGF2	98	PON1
3	ADH1C	35	CXCL10	67	IGFBP3	99	PPARA
4	ADRA1B	36	CXCL11	68	IKBKB	100	PPARD
5	ADRA2A	37	CXCL2	69	IL10RA	101	PPARG
6	AHR	38	CXCL8	70	IL1A	102	PRKCA
7	AKR1C3	39	CYP1A1	71	IL1B	103	PRKCB
8	AKT1	40	CYP1A2	72	IL2RA	104	PRKCD
9	ALOX5	41	CYP1B1	73	IL6R	105	PRSS1
10	AR	42	CYP3A4	74	IRF1	106	PTGS1
11	BAX	43	DPEP1	75	JUN	107	PTGS2
12	BCL2	44	DPP4	76	KDR	108	RAF1
13	BCL2L1	45	DUOX2	77	MAPK1	109	RASSF1
14	BIRC5	46	E2F1	78	MAPK8	110	RB1
15	CA2	47	EGF	79	MMP1	111	RELA
16	CASP3	48	EGFR	80	MMP2	112	RUNX2
17	CASP8	49	ERBB2	81	MMP3	113	SELE
18	CASP9	50	ERBB3	82	MMP9	114	SERPINE1
19	CAT	51	ESR1	83	MPO	115	SLC6A4
20	CAV1	52	ESR2	84	MYC	116	SLPI
21	CCL2	53	F3	85	NCF1	117	SOD1
22	CCNB1	54	FASN	86	NFE2L2	118	SPP1
23	CCND1	55	FOS	87	NFKBIA	119	STAT1
24	CD40LG	56	GJA1	88	NOS2	120	THBD
25	CDK1	57	GSK3B	89	NOS3	121	TNFAIP6
26	CDK2	58	GSTM1	90	NQO1	122	TOP1
27	CDKN1A	59	GSTP1	91	NR1I2	123	TOP2A
28	CHEK1	60	HIF1A	92	NR3C2	124	TP53
29	CHEK2	61	HMOX1	93	ODC1	125	VCAM1
30	CHUK	62	HSPA5	94	PARP1	126	VEGFA
31	CLDN4	63	HSPB1	95	PCNA		
32	COL1A1	64	ICAM1	96	PGR		

### Pharmacological network construction

**FIGURE 3:**
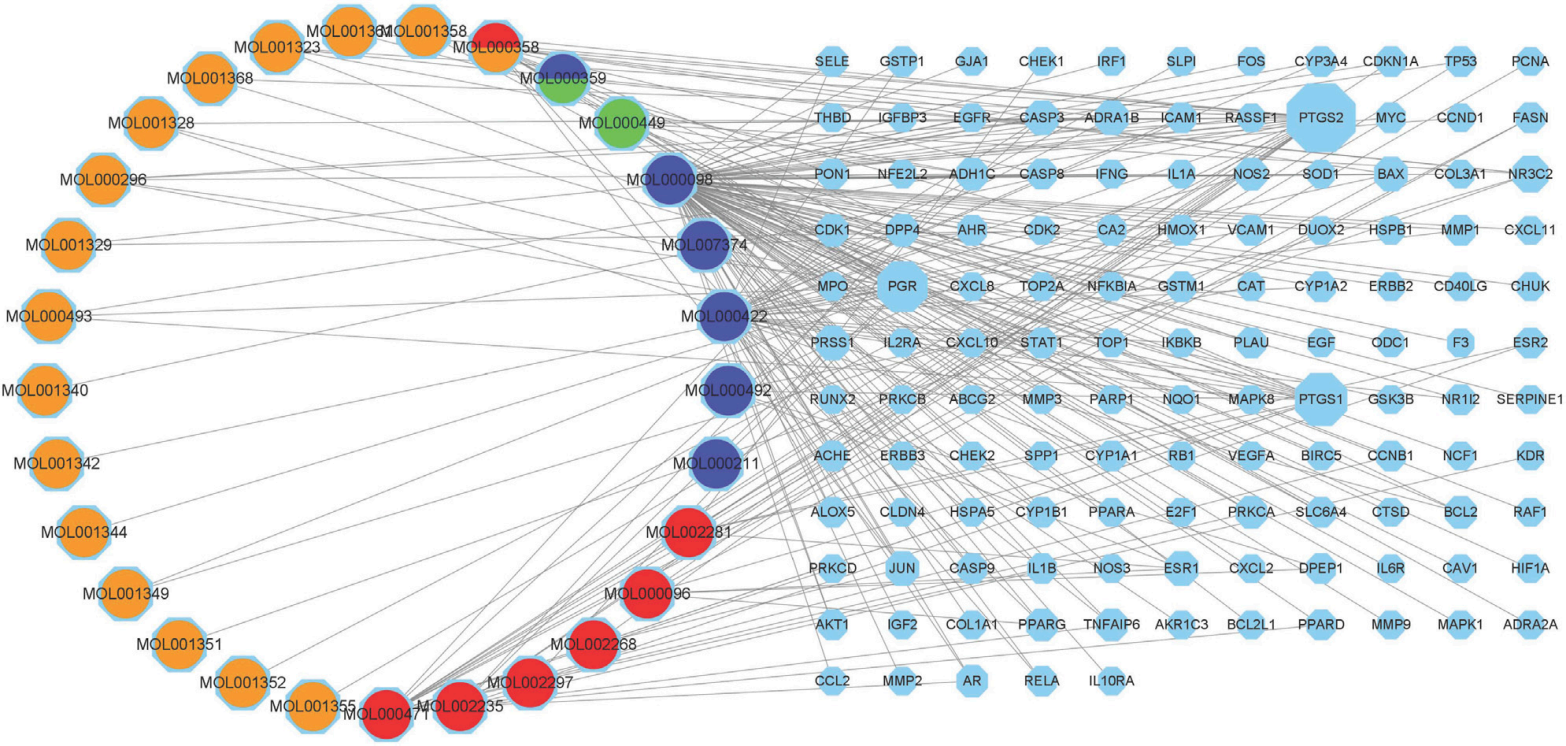
Composition-target network of RPD. This network shows the targeted relationship between the active components of TCM and the intersection of genes. Octagons represent targets. The left Circles stand for compound components. Orange represents tao ren, red represents da huang, blue represents dan pi, and green represents dong gua zi. MOL000358 stands for tao ren and da huang. MOL000359 belongs to dan pi and dong gua zi.

The targeting relationship between TCM active ingredients and intersection genes is presented by the TCM compound-disease regulatory network (Figure 3). Based on the “one to multiple,” and “multiple to one” links, the network possessed 155 nodes and 242 edges. The more nodes and edges acquired, the stronger the node interaction. Core genes possessed a higher degree which was positively correlated with node size and edge number. Active ingredients varied in different colors, orange represents tao ren, red represents da huang, blue represents dan pi, and green represents dong gua zi. Quercetin (MOL000098, degree = 103), kaempferol (MOL000422, degree = 37), aloe-emodin (MOL000471, degree = 15), beta-sitosterol (MOL000358, degree = 13), and (+)-catechin (MOL000096, degree = 6) were the top compounds and may be the critical nodes in the network, indicating greater significance anti-UC and IBS effect **(** Supplementary Table S2). Da huang and dan pi possessed a higher degree value, which is the main medicine in this prescription.

### PPI network and topological analysis

**FIGURE 4:**
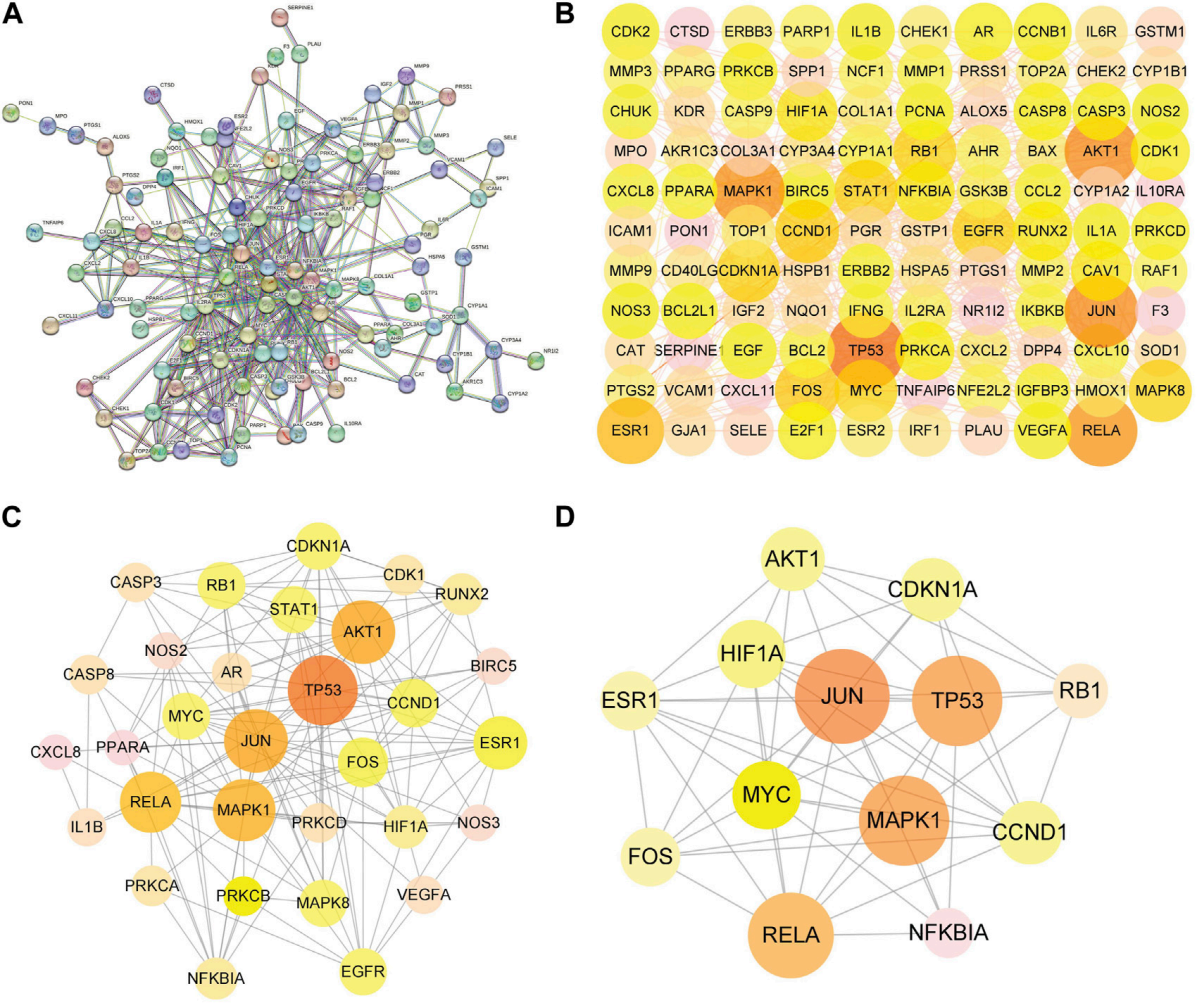
Protein–protein interaction (PPI) network of RPD against IBS and UC. **(A)** The interactive PPI network acquired from STRING database platform. **(B)** PPI network imported from STRING database to Cytoscape 3.8.0. **(C)** The PPI network of more significant proteins is composed of 31 nodes and 153 edges. **(D)** Core PPI network containing 13 nodes and 56 edges. A node with a larger size and deeper color possesses a higher degree value, such as JUN, TP53, MAPK1, RELA, and MYC.

The PPI network was constructed to clarify the relationship between RPD and diseases via the STRING software platform, with “the highest confidence (0.9)” for the minimum interaction threshold (Figure 4A). To further visualize and clarify the interactions of proteins, a new PPI network (Figure 4B) comprising 109 nodes and 433 connected edges was performed in Cytoscape 3.8.0. The average node Degree value is 15.89, and the average center clustering coefficient is 0.391. The width of the lines in the interactive PPI network represented the connection between the genes. Subsequently, the CytoNCA plugin was utilized for mining the core goals. The selection criteria were set based on corresponding median values as follows: BC >0, CC >0.4762, DC >6.00, EC >0.0527, NC >4.5714, and LAC >4.00. A new PPI network coinciding with the criteria was extracted with 31 nodes and 153 edges (Figure 4C). Likewise, the core PPI network that met the criteria (BC >13.837, CC >0.6, DC >20.00, EC >0.1645, NC >12.7178, and LAC >10.5455) was screened out comprising 13 nodes and 56 edges (Figure 4D). The size of the node represented the degree of the node in the interactive PPI network, with larger nodes and deeper color meaning higher degree values. The top nodes possessing higher degrees were regarded as hub genes, containing JUN (*n* = 40), TP53 (*n* = 38), MAPK1 (*n* = 38), RELA (*n* = 36), MYC (*n* = 28), and ESR1 (*n* = 24) (Supplementary Table S3). These target proteins were prepared to be docked with the main active ingredients analyzed earlier.

### GO biological processes of common target genes and KEGG pathway analysis

**FIGURE 5:**
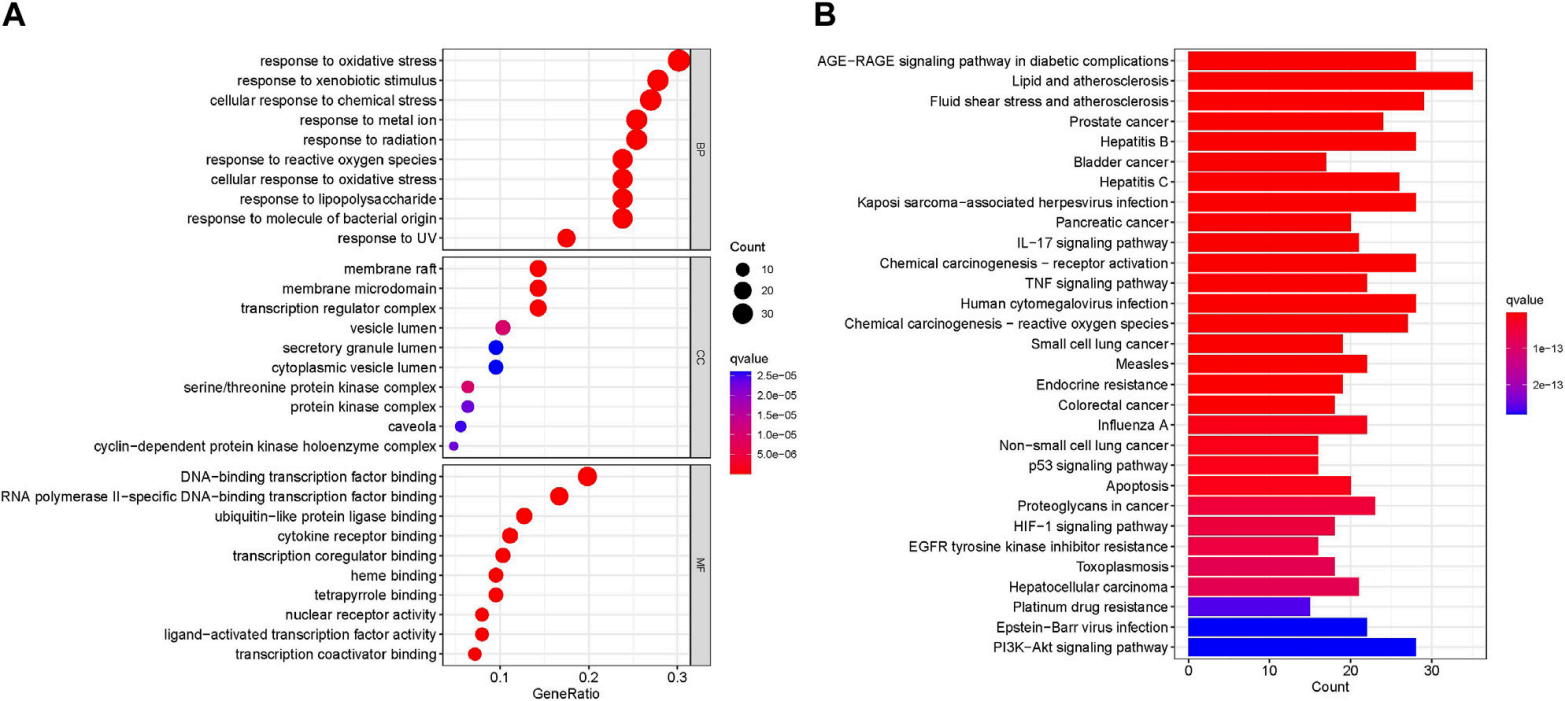
Enrichment Analysis **(A)** Bubble plot of GO enrichment analysis including the top 10 significant enrichment terms of three domains: BP, CC, and MF. The x-axis represents the gene proportion enriched in each entry, and y-axis shows the enrichment degree according to the q-value. **(B)** Barplot of KEGG enrichment pathways. The horizontal axis represents the number of target genes enriched in each entry, and the vertical axis shows the enrichment degree according to the corrected q-value. The redder bar means lower q value.

GO biological processes and KEGG pathway enrichment analysis were carried out to elucidate the functions and the enriched pathways of the potential treatment genes of RPD. The GO analysis consists of biological process (BP), cellular component (CC), and molecular function (MF). In this study, a total of 3,963 statistically significant GO terms were obtained with q-value <0.05, including 2053 of BP, 54 of CC, and 164 of MF. The top 10 terms were visualized in a bubble chart (Figure 5A). The redder the color of a dot, the lower the q-value and the greater the enrichment of the GO term. The results showed that response to oxidative stress (GO:0006979), response to xenobiotic stimulus (GO:0009410), and cellular response to chemical stress (GO:0062197) were tightly related to UC and IBS biological processes. The main disease-related terms in cellular components consisted of membrane raft (GO:0045121), membrane microdomain (GO:0098857), and transcription regulator complex (GO:0005667). Concerning molecular functions, more significant enrichment was found in DNA-binding transcription factor binding (GO:0140297), RNA polymerase II-specific DNA-binding transcription factor binding (GO:0061629), and ubiquitin-like protein ligase binding (GO:0044389).

KEGG analysis concentrated on the anti-IBS and anti-UC signaling pathways of RPD. The results demonstrated 166 statistically significant relative pathways, the top 20 significant enrichment potential pathways with the highest gene counts were presented in a barplot diagram **(** Figure 5B **)**, including “AGE-RAGE signaling pathway in diabetic complications,” “Lipid and atherosclerosis,” and “Fluid shear stress and atherosclerosis.” Additionally, five main pathways were involved in the treatment of common diseases by RPD: “NF-kappa B signaling pathway” (hsa04064), “MAPK signaling pathway” (hsa04010), “Inflammatory bowel disease” (hsa05321), and “Tp53 signaling pathway” (hsa04115) **(** Supplementary Table S4). Overall, these results indicated that RPD acts on the treatment of UC and IBS through multiple targets and multiple pathways involving inflammation, oxidative stress, and immunity. The KEGG map of MAPK and NF-kappa B signaling pathways is shown in Supplementary Figure S3.

### Molecular docking analysis

**FIGURE 6:**
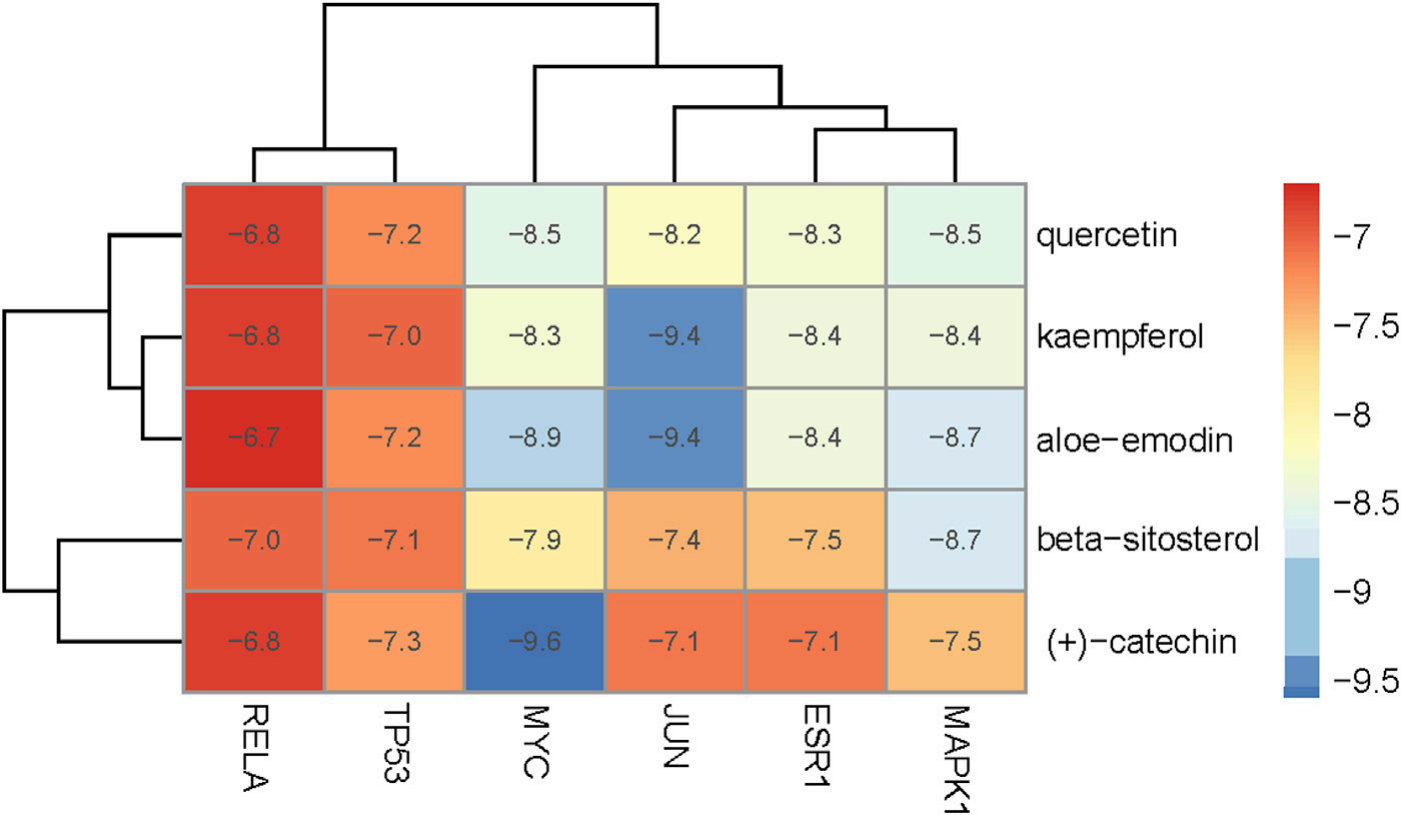
Heatmap based on binding energy among proteins and bioactives.

**FIGURE 7:**
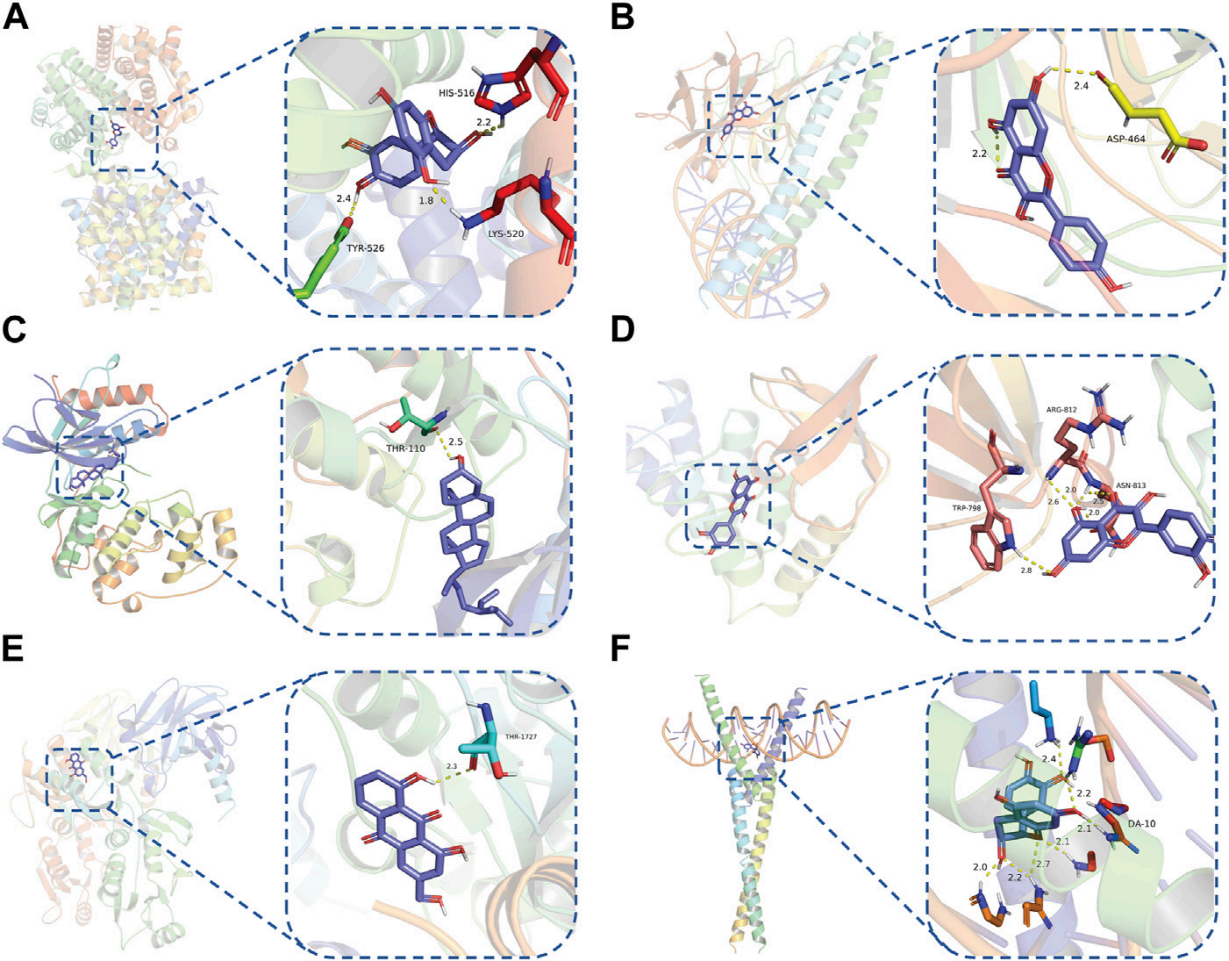
Binding mode of screened drugs to their targets by molecular docking: **(A)** ESR1- (+)-catechin; **(B)** JUN- kaempferol; **(C)** MAPK1-betasitosterol; **(D)** RELA- quercetin; **(E)** TP53- aloe-emodin; **(F)** MYC- (+)-catechin. Sticks represent ligands; the cartoon represents a hub target.

To evaluate the affinity of the candidate bioactive compounds for related targets, molecular docking analysis was performed for mimicking the interaction between small ligand molecules and receptor protein macromolecules. The binding poses and interactions of five ingredients with six proteins were obtained with Autodock Vina v.1.2.2. Additionally, the binding energy between the two counterparts for each interaction was calculated to predict their affinity (Table 3). Docking results indicated 3D interactions between small ligand molecules (chemical compounds) and receptor protein macromolecules in PyMOL software. The heatmap showed that the docking of MYC and (+)-catechin had the lowest binding energy (−9.6 kcal/mol), indicating that their stability was higher than the other combinations (Figure 6). Some local structures of molecular docking in detail were illustrated in Figure 7. The key active ingredients of RPD can bind well with key target genes, including ESR1 ((+)-catechin) (Figure 7A), JUN (kaempferol) (Figure 7B), MAPK1 (beta-sitosterol) (Figure 7C), RELA (quercetin) (Figure 7D), TP53 (aloe-emodin) (Figure 7E), and MYC- (+)-catechin (Figure 7F). This shows that good binding activity might play an important role in the treatment of UC and IBS. The above docking results suggested that they exert significant antioxidant and anti-inflammatory effects in the function of RPD.

**TABLE 3:** Docking results of five bioactive ingredients from RPD for IBS and UC targets.

Ingredients structure		Binding energy (kcal/mol)					
		JUN	TP53	MAPK1	RELA	MYC	ESR1
		1a02	1gzh	6g54	2vge	5i50	3os8
Quercetin	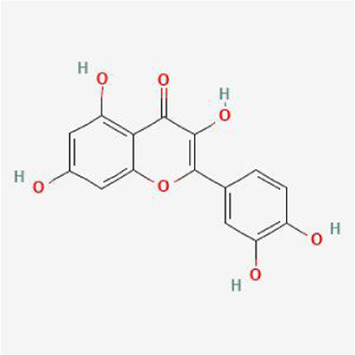	−8.2	−7.2	−8.5	−6.8	−8.5	−8.3
aloe-emodin	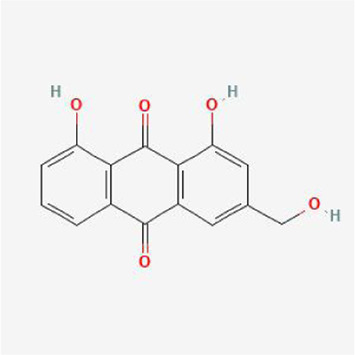	−9.4	−7.2	−8.7	−6.7	−8.9	−8.4
Kaempferol	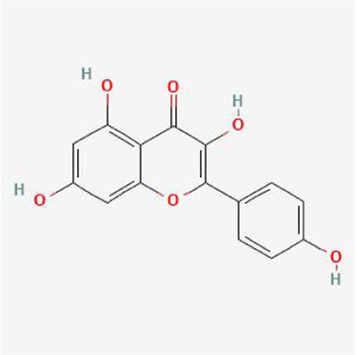	−9.4	−7.0	−8.4	−6.8	−8.3	−8.4
beta-sitosterol	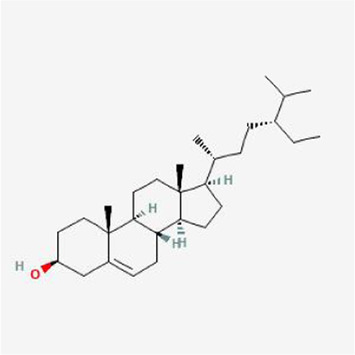	−7.4	−7.1	−8.7	−7.0	−7.9	−7.5
(+)-catechin	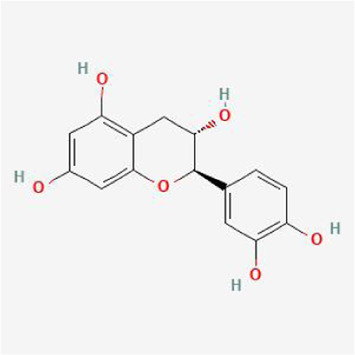	−7.1	−7.3	−7.5	−6.8	−9.6	−7.1

## Discussion

Although the significant difference between UC and IBS is the endoscopic manifestation, more than 30% of quiescent UC patients report IBS-like symptoms, irrespective of whether they are accompanied by low-grade inflammation or not. UC concurrent IBS tends toward more severe psychological symptoms and worse quality of life, so proper intervention is warranted [25, 26]. Despite various established treatments for UC or IBS, promising feasible therapies for the overlapping symptoms remains a challenge. It still represents a therapeutic conundrum in the clinic because of the lack of comprehensive guidelines. As chronic intestinal inflammation exerts multi-system effects with heterogeneous symptoms and numerous complications, long-term western medicine treatment facesd problems such as gradual reduction of efficacy and many side effects, and more drug targets need to be explored.

Guided by a holistic view and syndrome differentiation, TCM achieves multi-target and multi-pathway efficacy through prescription and prescription coordination and interaction. Network pharmacology is an excellent tool to construct a drug-target network and reveal the underlying mechanisms of TCM. Spleen deficiency was regarded as the original cause of UC based on TCM theory [27]. Additionally, spleen deficiency and liver stagnation are the root causes of IBS [28, 29]. There is a certain internal relationship between the constitution and the syndrome, so the same treatment can be adopted. Nowadays, RPD has extensively been used for UC in previous studies, and its specific effectiveness was acknowledged in improving intestinal mucous lesions, modifying gut microbiota dysbiosis, and reducing inflammatory response [14, 30]. However, there is a lack of holistic and systematic understanding of the material basis and molecular pharmacological mechanism of its efficacy. Therefore, we performed a multi-level network to further clarify the underlying regulatory mechanism of RPD in the process of UC and IBS, thus enabling the provision ofadequate recommendations regarding potential treatment options.

By integrating all the targets of RPD and disease, 126 relative targets were identified, and top targets such as JUN, TP53, MAPK1, RELA, MYC, and ESR1 in the PPI network were considered as hub genes. Meanwhile, the GO and KEGG analysis revealed that inflammation, oxidative stress, immunity, oncogenicity, and gut microbiota dysbiosis are significantly involved in the pathological mechanisms of UC and IBS. BP enrichment results indicated that a variety of genes were associated with oxidative stress, including “response to oxidative stress,” “response to xenobiotic stimulus,” and “cellular response to chemical stress.” Emerging evidence has made great strides in clarifying that oxidative stress plays a crucial role in the development of colitis [31]. During inflammatory episodes, diffusive inflammatory cell infiltration and small intestinal mucosal crypt abscesses in colitis trigger the over-production of reactive oxygen species (ROS), followed by intestinal cell layer damage via free radical-dependent apoptosis. These pathological states lead to pathogen invasion and exaggeration of inflammatory cell infiltration as well as inflammatory damage [32], which ultimately promotes the initiation and progression of UC. Furthermore, several studies associate mild inflammation with IBS, and oxidative stress may be a crucial pathological factor since tissue damage due to oxidative attack is currently associated with inflammation [33, 34]. Moreover, an extensive study reported shreds of evidence of immune activation and epithelial barrier dysfunction in IBS patients with no other features of overt inflammation, containing intraepithelial lymphocyte aggregation and proinflammatory cytokine expression [35]. Therefore, ROS-targeted treatment and antioxidant prevention were proposed to be potential measures for alleviating UC and IBS [36].

Furthermore, the molecular docking results indicate that MAPK1, RELA, TP53, JUN, MYC, and ESR1, have superior affinities to the five core compounds quercetin, kaempferol, aloe-emodin, beta-sitosterol and (+)-catechin, which verified that colon damage may be repaired by improving inflammation, and reducing oxidative stress. Quercetin is a natural polyphenolic compound belonging to the flavonoid family. It is considered the most effective reactive oxygen species (ROS) scavenger by upregulating proinflammatory factors. Multiple animal models have confirmed quercetin exerts antioxidant and anti-inflammatory effects [37] by appearing to be mediated via interference in signal transduction pathways including the NF-κB and kinase systems [38]. Quercetin could offer dose-dependent protection for colitis mice through an antioxidant-like effect [39]. It is congruent with the fact that inflammatory responses are the important pathological mechanism of UC and IBS. Kaempferol is also a flavonoid with anti-inflammatory effects and has potential effects on alleviating murine experimental colitis by inhibiting the LPS-TLR4-NF-κB Axis and altering gut microbiota [40].

Likewise, aloe-emodin (a bioactive component of rhubarb), with a higher degree value in the PPI network, exerted an anti-inflammatory role by suppressing the secretion of inflammatory factors, downregulating the cascade reaction of NF-κB, MAPK, and PI3K pathways [41]. Aloe vera improved UC by enhancing colon mucus barrier functions in addition to reducing inflammation [42]. Increasing experimental and clinical evidence demonstrated that emodin exerted multi-targeting therapeutic mechanisms, involving anti-inflammatory, immunomodulatory, anti-fibrosis, and anti-tumor properties [15]. Catechins-flavanols attributes to flavonoids with diverse biological functions. They could exert potent antioxidant effects by inducing apoptosis, arresting cell-cycle, inhibiting NF-κB, and suppressing COX-2 overexpression *in vitro* and animal experiments [43]. β-Sitosterol is a phytosterol in plants that may play an anti-inflammatory role by reducing the production of proinflammatory cytokine and ROS [44]. Kang et al. found that novel effects of β-Sitosterol contributed to improving UC by exerting antimicrobial activity [45]. Taken together, the bioactive ingredients screened from RPD played significant roles in anti-inflammatory properties. These pharmacological activities indicated the interaction between “multiply compounds,” “multiply targets,” and “multiply pathways” of RPD.

KEGG analysis results demonstrated that the mechanism of RPD in the treatment of UC and IBS was mainly related to the following pathways indicating multiple targets and multiple pathways: “AGE-RAGE signaling pathway in diabetic complications,” “Inflammatory bowel disease,” “NF-κB signaling pathway,” “MAPK signaling pathway” and “Tp53 signaling pathway.” AGE-RAGE signaling pathway was involved in and mediated various signaling pathways by activating the PI3K-Akt and MAPK as well as the NF-κB signaling pathway, to induce ROS production and lead to inflammatory responses. NF-κB is acknowledged as a sign and the central pathway of inflammatory response, mediating various signaling pathways of inflammatory response [46]. The transcription factor NF-κB initiates expression of proinflammatory cytokines including IL-6, TNF-α, and IFN-γ [47]. The polymorphism of NFKB1A is associated with an increased risk of UC [48]. RELA is also a crucial member of the NF-κB family. Saito et. al claimed that compared to the resting proximal colon, a higher methylation level of ESR1 was detected in the actively inflamed colon [49]. Advanced glycosylation end products (AGE) and IL-17 can also be used as mediators for NF-κB pathway activation [50, 51]. Higher RAGE levels and RAGE polymorphisms have been found in UC patients correlated with the nuclear expression of pro-inflammatory, pro-oxidant, and pro-oncogenic genes [52], thus these pathways may be the potential inhibition point of intestinal symptoms and the progression to colorectal cancer (CRC) [47].

It is well-known that CRC is associated with the inflammation–dysplasia sequence, which poses a major threat to the prognosis of UC and IBS. The incidence of UC-related colon cancers was 1.6∼3.7%, accounting for 5.4% in all colon types [53, 54]. Chronic inflammation, the increased turnover of epithelial cells, and ROS production are thought to contribute to the development of dysplastic lesions, which may then transform into CRC [55]. JUN is a proto-oncogene that can induce oncogenic transformation [56]. The mutation of the p53 gene is a critical genetic change, involved in the early stages of UC-associated carcinogenesis of the colorectum. It is also used as a biomarker in predicting the risk of evolution toward malignancy [57].

Gut microbiota alternation is one of the underlying factors in perpetuating IBS symptoms [58]. Both our study and another study confirmed that the action mechanism of RPD is related to microbiota [14]. RPD could restore α diversity by enhancing *Firmicutes* and *Actinobacteria* and reducing *Proteobacteria* and *Bacteroidetes* [14]. Furthermore, quercetin was verified to exert a therapeutic effect in UC by altering the composition of gut microbiota [59]. The abundance of *Bifidobacterium*, *Bacteroides*, and *Lactobacillus* increased significantly while *Fusobacterium* and *Enterococcus* decreased totally. These findings indicate that RPD has the comprehensive ability to modify gut microbiota and suppress pro-inflammatory cytokines. Therefore, our results indicate that RPD may also have a role in regulating intestinal microbiota.

UC is a chronic recurrent intestinal inflammatory disease, whereas IBS is a functional intestinal disorder. The current clinical treatments for them are different, as IBS requires mainly symptomatic treatments. And the goal of UC treatment is to achieve clinical or endoscopic remission. It is challenging to tell the difference between IBS patients and UC in remission due to the numerous similarities between the two. Timely and accurate identification is key to avoiding overtreatment of IBS or delayed diagnosis of UC. When the two conditions are difficult to distinguish, Chinese medicine may be a proper option. However, the present study has several limitations. Firstly, absorption pathways, effective sites, and dose-effect outcomes, markedly effective components of bioactives in RPD, should be studied in depth. Secondly, the complex interaction between gut microbiota and herbal compounds needs more exploration. Moreover, any single certain bioactive detected in RPD is not sufficient to stand in completely for RPD. Thus, more experiments are required to further explore the potential molecular mechanism of RPD in the treatment of UC-IBS *in vitro* and *in vivo*.

## Conclusion

In conclusion, both network pharmacology and molecular docking were applied to predict the common mechanism of RPD for the treatment of UC and IBS. The GO and KEGG pathways significantly enriched several signaling pathways involved in inflammation, oxidative stress, immunity, oncogenicity, and gut microbiota dysbiosis. Meanwhile, molecular docking results further verified that all the key active ingredients exhibited superior affinities to their core targets. RPD has revealed synergistic therapeutic effects in the treatment of UC and IBS partly by regulating multi-component, multi-target, and multi-pathway effects, which coincide with the biological mechanism of “treating different diseases with the same treatment.” This study offers novel insights into the effective therapeutic and management strategies for patients overlapping between UC and IBS, proposing future directions for research in this stimulating field.

## Data Availability

The original contributions presented in the study are included in the article/Supplementary Material, further inquiries can be directed to the corresponding author.

## Abbreviations

IBS, Irritable Bowel Syndrome; UC, Ulcerative Colitis; IBD, Inflammatory bowel disease; RPD, Rhubarb peony decoction; KEGG, Kyoto Encyclopedia of Genes and Genomes; GO, Gene Ontology; PPI, Protein-Protein Interaction; MAPK, Mitogen-activated protein kinase; OB, Oral bioavailability; DL, Drug likeness.
