# Supplementary material for: Network pharmacology and molecular docking reveal the immunomodulatory mechanism of rhubarb peony decoction for the treatment of ulcerative colitis and irritable bowel syndrome
Source: J Pharm Pharm Sci. 2023 May 25;26:11225. doi: 10.3389/jpps.2023.11225 (PMC10248730; doi:10.3389/jpps.2023.11225)
Supplement: Supplementary file 1 [file DataSheet1.docx]

Supplementary Material

# **Supplementary Figures and Tables**

## Supplementary Figures

**
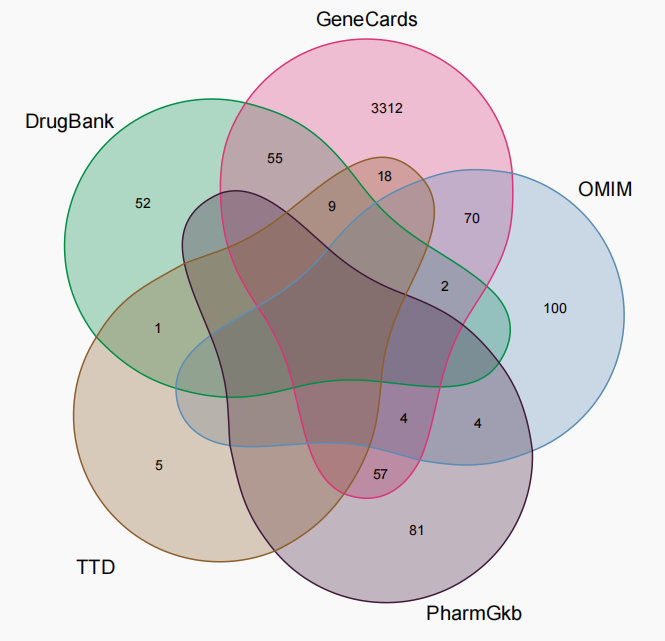
**

**Supplementary Figure S1.** **Venn diagram of disease targets for IBS**

**
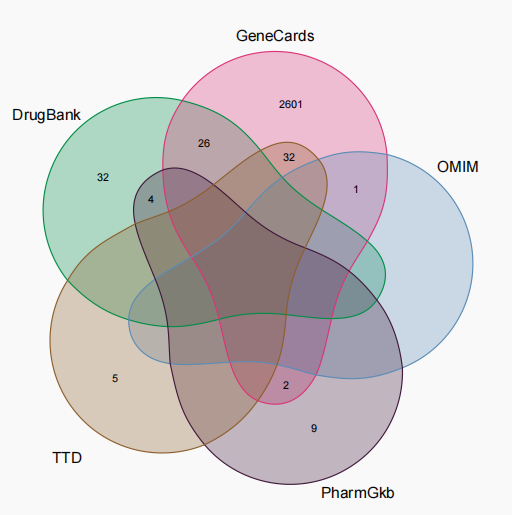
**

**Supplementary Figure S2. Venn diagram of disease targets for UC**

**
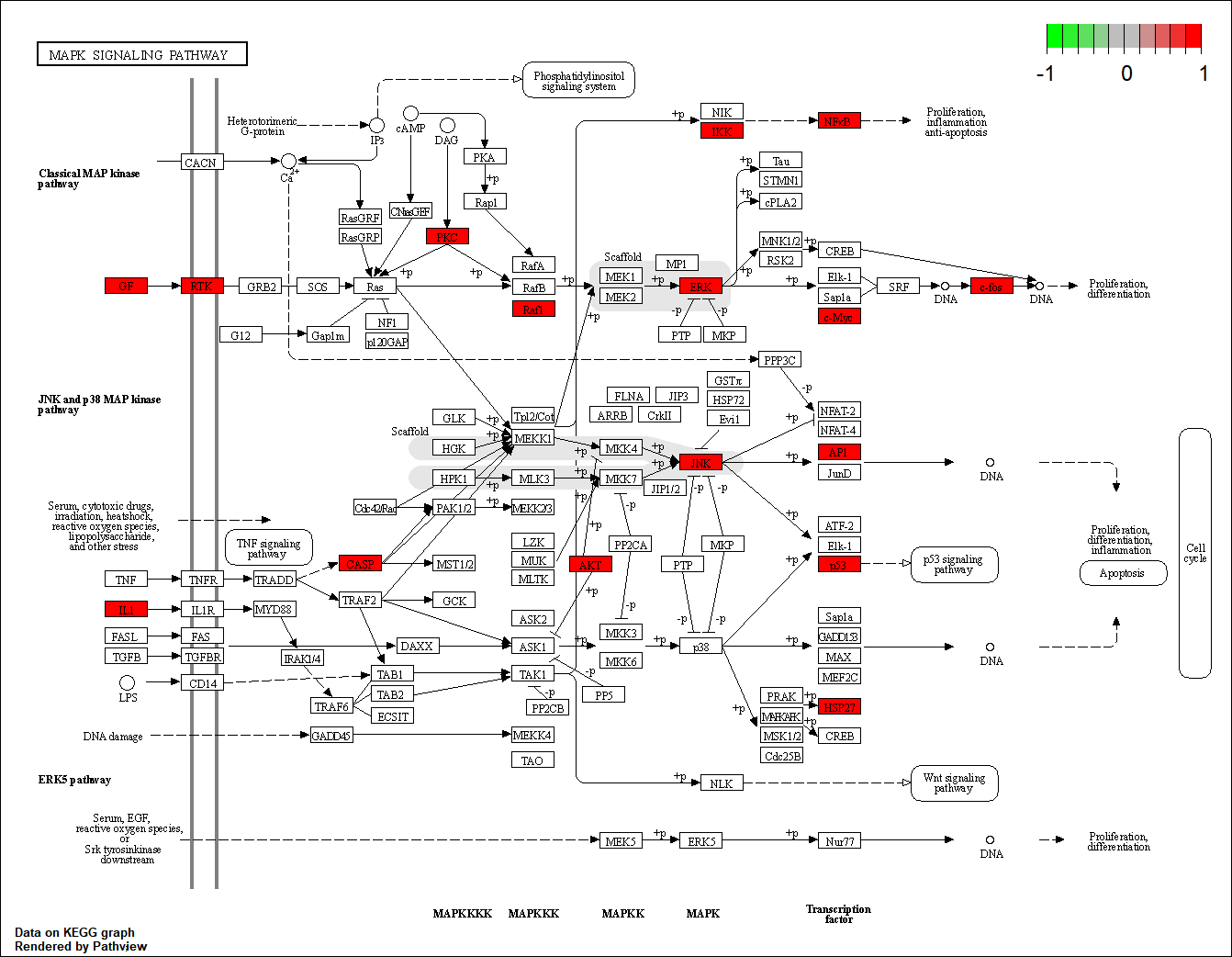
**

**Supplementary Figure S3(A)The KEGG map of MAPK signaling pathway (hsa04010)**

**
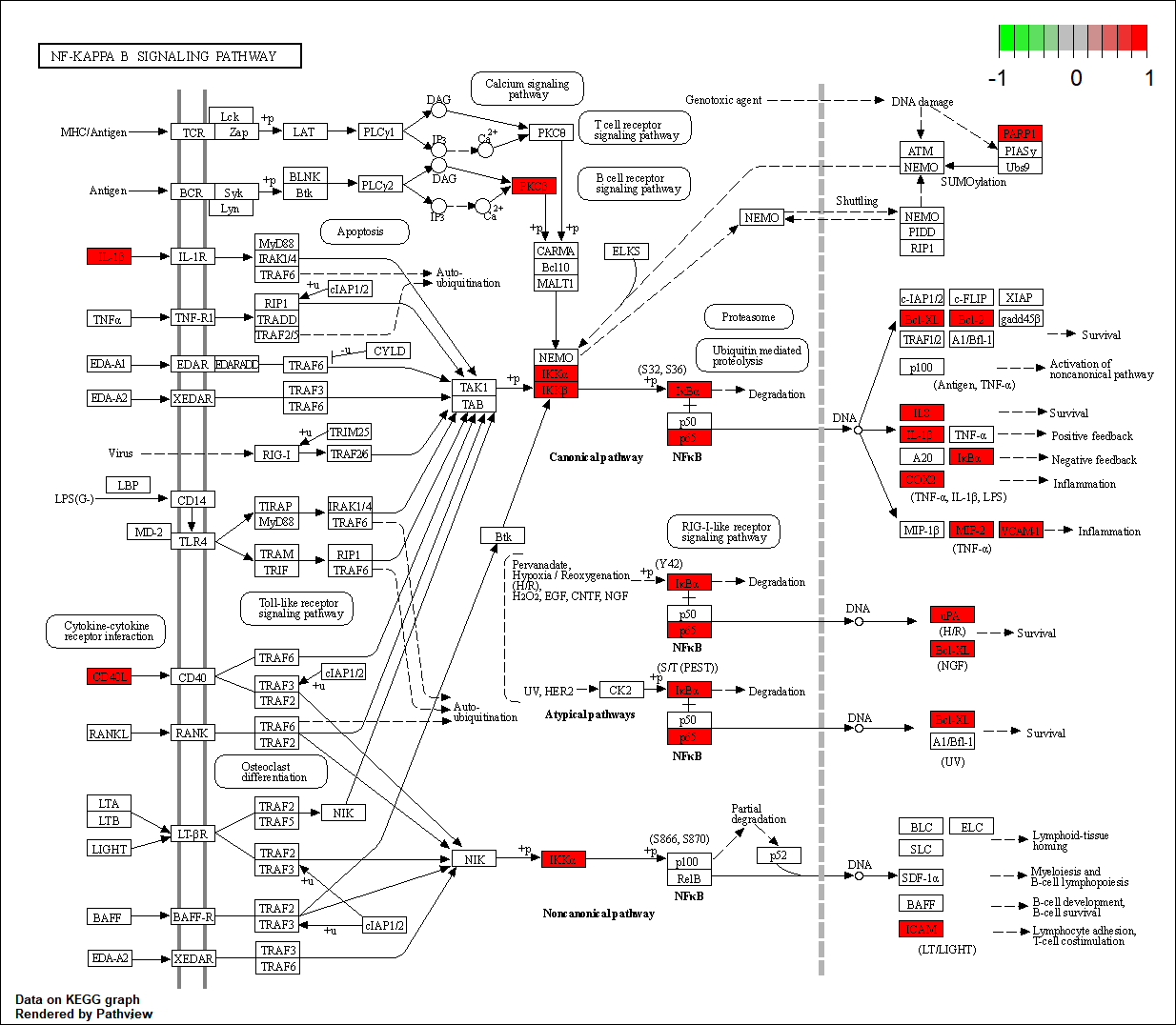
**

**Supplementary Figure S3(B) The KEGG map of** **NF-kappa B signaling pathway (hsa04064)**

## 1.2 Supplementary Tables

**Supplementary Table S1 Active compounds of** **Rhubarb Peony Decoction**

| Drug | Mol Id | Molecule Name | Symbol | OB (%) | DL |
| --- | --- | --- | --- | --- | --- |
| Dahuang | MOL002235 | EUPATIN | NOS2 | 50.8 | 0.41 |
| Dahuang | MOL002268 | rhein | PTGS1 | 47.07 | 0.28 |
| Dahuang | MOL002281 | Toralactone | NOS2 | 46.46 | 0.24 |
| Dahuang | MOL002297 | Daucosterol_qt | PGR | 35.89 | 0.7 |
| Dahuang | MOL000358 | beta-sitosterol | PGR | 36.91 | 0.75 |
| Dahuang | MOL000471 | aloe-emodin | PTGS1 | 83.38 | 0.24 |
| Dahuang | MOL000096 | (-)-catechin | PTGS1 | 49.68 | 0.24 |
| Danpi | MOL000211 | Mairin | PGR | 55.38 | 0.78 |
| Danpi | MOL000359 | sitosterol | PGR | 36.91 | 0.75 |
| Danpi | MOL000422 | kaempferol | NOS2 | 41.88 | 0.24 |
| Danpi | MOL000492 | (+)-catechin | PTGS1 | 54.83 | 0.24 |
| Danpi | MOL007374 | 5-[[5-(4-methoxyphenyl)-2-furyl]methylene]barbituric acid | ESR1 | 43.44 | 0.3 |
| Danpi | MOL000098 | quercetin | PTGS1 | 46.43 | 0.28 |
| Dongguazi | MOL000449 | Stigmasterol | PGR | 43.83 | 0.76 |
| Taoren | MOL001323 | Sitosterol alpha1 | PGR | 43.28 | 0.78 |
| Taoren | MOL001328 | 2,3-didehydro GA70 | PTGS1 | 63.29 | 0.5 |
| Taoren | MOL001329 | 2,3-didehydro GA77 | PTGS2 | 88.08 | 0.53 |
| Taoren | MOL001340 | GA120 | CHRM3 | 84.85 | 0.45 |
| Taoren | MOL001342 | GA121-isolactone | PGR | 72.7 | 0.54 |
| Taoren | MOL001344 | GA122-isolactone | PGR | 88.11 | 0.54 |
| Taoren | MOL001349 | 4a-formyl-7alpha-hydroxy-1-methyl-8-methylidene-4aalpha,4bbeta-gibbane-1alpha,10beta-dicarboxylic acid | NR3C2 | 88.6 | 0.46 |
| Taoren | MOL001351 | Gibberellin A44 | NR3C2 | 101.61 | 0.54 |
| Taoren | MOL001352 | GA54 | PTGS2 | 64.21 | 0.53 |
| Taoren | MOL001353 | GA60 | CHRM2 | 93.17 | 0.53 |
| Taoren | MOL001355 | GA63 | PTGS2 | 65.54 | 0.54 |
| Taoren | MOL001358 | gibberellin 7 | CHRM3 | 73.8 | 0.5 |
| Taoren | MOL001360 | GA77 | GABRA1 | 87.89 | 0.53 |
| Taoren | MOL001361 | GA87 | PTGS2 | 68.85 | 0.57 |
| Taoren | MOL001368 | 3-O-p-coumaroylquinic acid | PTGS1 | 37.63 | 0.29 |
| Taoren | MOL000296 | hederagenin | PGR | 36.91 | 0.75 |
| Taoren | MOL000493 | campesterol | PGR | 37.58 | 0.71 |

**Supplementary Table S2 The intersection targets of RPD and disease of IBS and UC**

| Mol Id | Degree | Mol Id | Degree |
| --- | --- | --- | --- |
| MOL000098 | 103 | MOL001368 | 2 |
| MOL000422 | 37 | MOL001361 | 2 |
| MOL000471 | 15 | MOL001358 | 2 |
| MOL000358 | 13 | MOL001349 | 2 |
| MOL000449 | 8 | MOL001329 | 2 |
| MOL002235 | 8 | MOL000359 | 2 |
| MOL000096 | 6 | MOL001355 | 1 |
| MOL002281 | 6 | MOL001352 | 1 |
| MOL000296 | 5 | MOL001351 | 1 |
| MOL000492 | 5 | MOL001344 | 1 |
| MOL001323 | 4 | MOL001342 | 1 |
| MOL000493 | 3 | MOL001340 | 1 |
| MOL001328 | 3 | MOL000211 | 1 |
| MOL007374 | 3 | MOL002297 | 1 |
| MOL002268 | 3 |  |  |

**Supplementary Table S3 Information of the key active genes of RPD in the treatment of UC and IBS**

| Name | Betweenness | Closeness | Degree | Eigenvector | Information | LAC | Network | Selected |
| --- | --- | --- | --- | --- | --- | --- | --- | --- |
| JUN | 118.249484 | 0.75 | 40 | 0.30413777 | 12.1078491 | 14.4 | 28.1555721 | FALSE |
| TP53 | 76.8652639 | 0.73170732 | 38 | 0.30031258 | 11.9387112 | 14.3157895 | 25.2675419 | FALSE |
| MAPK1 | 84.8875701 | 0.73170732 | 38 | 0.28487965 | 11.9387112 | 13.2631579 | 25.5037945 | FALSE |
| RELA | 93.1052326 | 0.71428571 | 36 | 0.26318163 | 11.7567234 | 12.4444444 | 23.6652474 | FALSE |
| MYC | 21.3059273 | 0.65217391 | 28 | 0.25582775 | 10.8657341 | 15.1428571 | 19.0722679 | FALSE |
| HIF1A | 31.3572498 | 0.65217391 | 28 | 0.24594823 | 10.8657341 | 14 | 18.5131097 | FALSE |
| CDKN1A | 23.8691503 | 0.625 | 26 | 0.21432982 | 10.5907612 | 11.3846154 | 14.5047638 | FALSE |
| AKT1 | 25.2763237 | 0.6122449 | 26 | 0.21224096 | 10.5907612 | 11.0769231 | 14.1407961 | FALSE |
| CCND1 | 21.7243525 | 0.63829787 | 26 | 0.22730887 | 10.5907612 | 13.2307692 | 16.7971613 | FALSE |
| FOS | 17.2662909 | 0.625 | 24 | 0.21276055 | 10.2887306 | 11.6666667 | 13.4983933 | FALSE |
| ESR1 | 13.8369841 | 0.625 | 24 | 0.22804189 | 10.2887306 | 13.6666667 | 15.1229457 | FALSE |
| RB1 | 17.2382386 | 0.6122449 | 22 | 0.18324099 | 9.95544052 | 10.5454545 | 12.8686191 | FALSE |
| NFKBIA | 14.4332351 | 0.6 | 20 | 0.16451436 | 9.58577633 | 10 | 12.7178459 | FALSE |

**Supplementary Table S4 The significantly enrichment potential pathways of KEGG enrichment analysis**

| ID | Signaling Pathway | Gene Ratio | P-value | P-adjust | Q-value | Gene ID |
| --- | --- | --- | --- | --- | --- | --- |
| hsa04933 | AGE-RAGE signaling pathway in diabetic complications | 28/120 | 3.41E-29 | 8.32E-27 | 2.80E-27 | AKT1/BAX/BCL2/CASP3/CCL2/CCND1/COL1A1/COL3A1/CXCL8/F3/ICAM1/IL1A/IL1B/JUN/MAPK1/MAPK8/MMP2/NOS3/PRKCA/PRKCB/PRKCD/RELA/SELE/SERPINE1/STAT1/THBD/VCAM1/VEGFA |
| hsa05207 | Chemical carcinogenesis- receptor activation | 28/120 | 1.37E-19 | 3.04E-18 | 1.02E-18 | AHR/AKT1/AR/BCL2/BIRC5/CCND1/CYP1A1/CYP1A2/CYP1B1/CYP3A4/E2F1/EGF/EGFR/ESR1/ESR2/FOS/GSTM1/JUN/MAPK1/MYC/PGR/PPARA/PRKCA/PRKCB/RAF1/RB1/RELA/VEGFA |
| hsa05208 | Chemical carcinogenesis- reactive oxygen species | 27/120 | 6.69E-18 | 1.17E-16 | 3.93E-17 | AHR/AKR1C3/AKT1/CAT/CHUK/CYP1A1/CYP1A2/CYP1B1/EGF/EGFR/FOS/GSTM1/HIF1A/HMOX1/IKBKB/JUN/MAPK1/MAPK8/NCF1/NFE2L2/NFKBIA/NQO1/PRKCD/RAF1/RELA/SOD1/VEGFA |
| hsa04010 | MAPK signaling pathway | 24/120 | 4.01E-12 | 2.39E-11 | 8.04E-12 | AKT1/CASP3/CHUK/EGF/EGFR/ERBB2/ERBB3/FOS/HSPB1/IGF2/IKBKB/IL1A/IL1B/JUN/KDR/MAPK1/MAPK8/MYC/PRKCA/PRKCB/RAF1/RELA/TP53/VEGFA |
| hsa05205 | Proteoglycans in cancer | 23/120 | 1.37E-14 | 1.46E-13 | 4.90E-14 | AKT1/CASP3/CAV1/CCND1/CDKN1A/COL1A1/EGFR/ERBB2/ERBB3/ESR1/HIF1A/IGF2/KDR/MAPK1/MMP2/MMP9/MYC/PLAU/PRKCA/PRKCB/RAF1/TP53/VEGFA |
| hsa04668 | TNF signaling pathway | 22/120 | 2.45E-19 | 4.99E-18 | 1.68E-18 | AKT1/CASP3/CASP8/CCL2/CHUK/CXCL10/CXCL2/FOS/ICAM1/IKBKB/IL1B/IRF1/JUN/MAPK1/MAPK8/MMP3/MMP9/NFKBIA/PTGS2/RELA/SELE/VCAM1 |
| hsa04657 | IL-17 signaling pathway | 21/120 | 9.86E-20 | 2.41E-18 | 8.10E-19 | CASP3/CASP8/CCL2/CHUK/CXCL10/CXCL2/CXCL8/FOS/GSK3B/IFNG/IKBKB/IL1B/JUN/MAPK1/MAPK8/MMP1/MMP3/MMP9/NFKBIA/PTGS2/RELA |
| hsa04210 | Apoptosis | 20/120 | 4.48E-15 | 4.97E-14 | 1.67E-14 | AKT1/BAX/BCL2/BCL2L1/BIRC5/CASP3/CASP8/CASP9/CHUK/CTSD/FOS/IKBKB/JUN/MAPK1/MAPK8/NFKBIA/PARP1/RAF1/RELA/TP53 |
| hsa05210 | Colorectal cancer | 18/120 | 1.75E-16 | 2.37E-15 | 7.96E-16 | AKT1/BAX/BCL2/BIRC5/CASP3/CASP9/CCND1/CDKN1A/EGF/EGFR/FOS/GSK3B/JUN/MAPK1/MAPK8/MYC/RAF1/TP53 |
| hsa04066 | HIF-1 signaling pathway | 18/120 | 1.45E-14 | 1.47E-13 | 4.96E-14 | AKT1/BCL2/CDKN1A/EGF/EGFR/ERBB2/HIF1A/HMOX1/IFNG/IL6R/MAPK1/NOS2/NOS3/PRKCA/PRKCB/RELA/SERPINE1/VEGFA |
| hsa04064 | NF-kappa B signaling pathway | 16/120 | 1.47E-12 | 9.20E-12 | 3.10E-12 | BCL2/BCL2L1/CD40LG/CHUK/CXCL2/CXCL8/ICAM1/IKBKB/IL1B/NFKBIA/PARP1/PLAU/PRKCB/PTGS2/RELA/VCAM1 |
| hsa04115 | p53 signaling pathway | 16/120 | 4.25E-15 | 4.94E-14 | 1.66E-14 | BAX/BCL2/BCL2L1/CASP3/CASP8/CASP9/CCNB1/CCND1/CDK1/CDK2/CDKN1A/CHEK1/CHEK2/IGFBP3/SERPINE1/TP53 |
| hsa04630 | JAK-STAT signaling pathway | 14/120 | 8.72E-08 | 2.73E-07 | 9.18E-08 | AKT1/BCL2/BCL2L1/CCND1/CDKN1A/EGF/EGFR/IFNG/IL10RA/IL2RA/IL6R/MYC/RAF1/STAT1 |
| hsa05321 | Inflammatory bowel disease | 6/120 | 0.00035595 | 0.00076 | 0.000254 | IFNG/IL1A/IL1B/JUN/RELA/STAT1 |
